# Brownian Dynamics Simulation of Nucleocytoplasmic Transport: A Coarse-Grained Model for the Functional State of the Nuclear Pore Complex

**DOI:** 10.1371/journal.pcbi.1002049

**Published:** 2011-06-02

**Authors:** Ruhollah Moussavi-Baygi, Yousef Jamali, Reza Karimi, Mohammad R. K. Mofrad

**Affiliations:** Molecular Cell Biomechanics Laboratory, Department of Bioengineering, University of California, Berkeley, California, United States of America; Stanford University, United States of America

## Abstract

The nuclear pore complex (NPC) regulates molecular traffic across the nuclear envelope (NE). Selective transport happens on the order of milliseconds and the length scale of tens of nanometers; however, the transport mechanism remains elusive. Central to the transport process is the hydrophobic interactions between karyopherins (kaps) and Phe-Gly (FG) repeat domains. Taking into account the polymeric nature of FG-repeats grafted on the elastic structure of the NPC, and the kap-FG hydrophobic affinity, we have established a coarse-grained model of the NPC structure that mimics nucleocytoplasmic transport. To establish a foundation for future works, the methodology and biophysical rationale behind the model is explained in details. The model predicts that the first-passage time of a 15 nm cargo-complex is about 2.6±0.13 ms with an inverse Gaussian distribution for statistically adequate number of independent Brownian dynamics simulations. Moreover, the cargo-complex is primarily attached to the channel wall where it interacts with the FG-layer as it passes through the central channel. The kap-FG hydrophobic interaction is highly dynamic and fast, which ensures an efficient translocation through the NPC. Further, almost all eight hydrophobic binding spots on kap-β are occupied simultaneously during transport. Finally, as opposed to intact NPCs, cytoplasmic filaments-deficient NPCs show a high degree of permeability to inert cargos, implying the defining role of cytoplasmic filaments in the selectivity barrier.

## Author Summary

Perforating and spanning the nuclear envelope (NE), the nuclear pore complex (NPC) is a supramolecular assembly that regulates all traffic between the nucleus and cytoplasm. As the unique gateway to the nucleus, NPC selectively facilitates the transport of large cargo while offering a relatively unobstructed pathway for small molecules and ions. Despite the high throughput of about 1000 translocations per NPC per second, the NPC strictly controls the passage of individual cargos. However, the dynamic mechanism of nucleocytoplasmic transport is poorly understood. It is too difficult to experiment on the transport mechanism within the confined geometry of this tiny pore *in vivo*. Currently, only computational techniques can elucidate the detailed events happening at this tiny pore with a refined spatiotemporal resolution to account for transient bonds. Based on experimental data regarding the NPC structure and nucleocytoplasmic transport, we have established a coarse-grained model of the functional state of the NPC. The model mimics nucleocytoplasmic transport and allows us to directly observe the processes happening within the pore from a biophysical perspective. The first-passage time of a single cargo-complex is found to be about 2.6 ms. Furthermore, kap-FG hydrophobic bonds are highly dynamic and short-lived, ensuring efficient transport.

## Introduction

**Figure 1:**
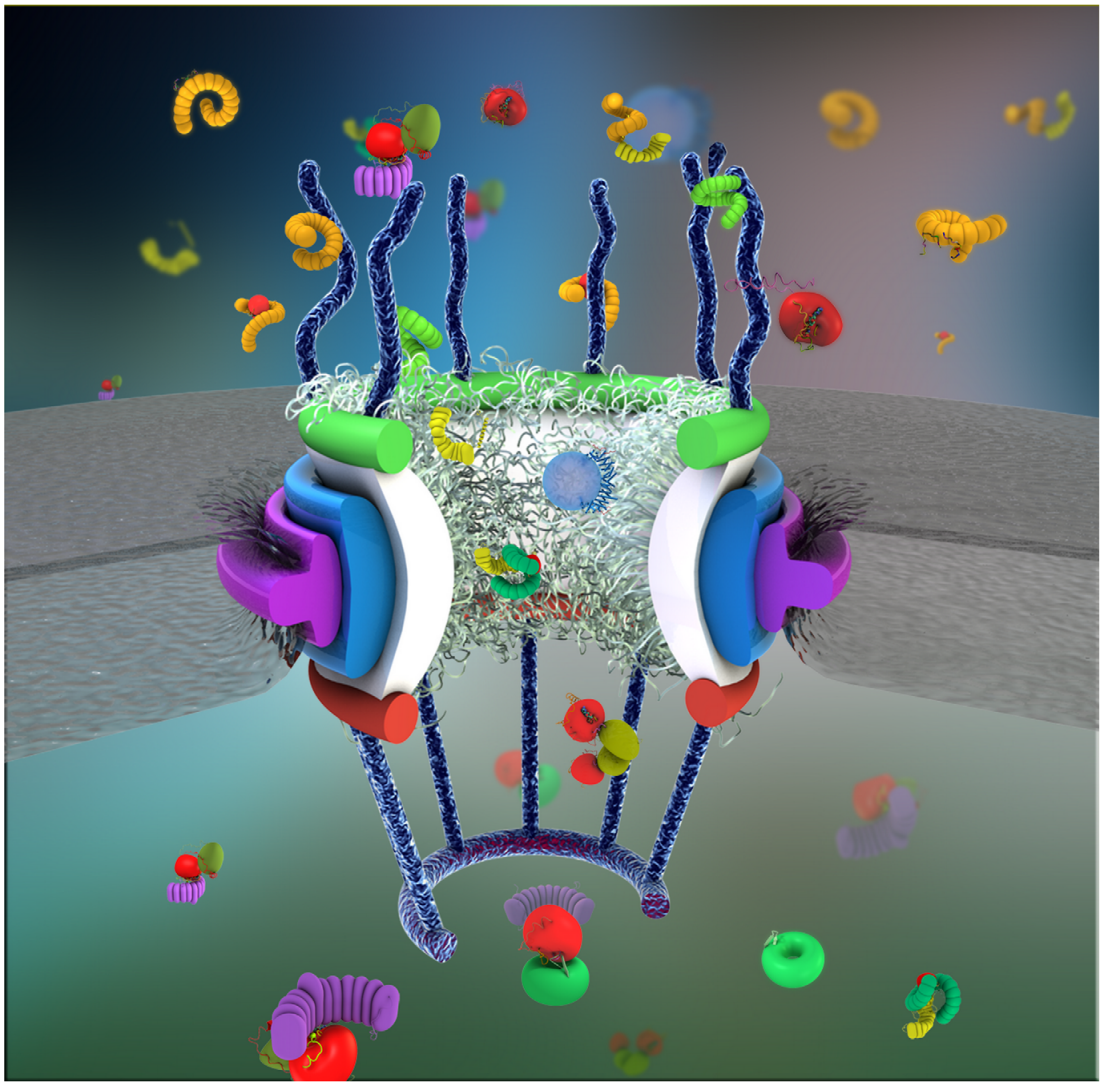
A schematic representation of the NPC structure with the cargo-complex indicated as a kap-β-bound blue sphere inside the central channel. For more excellent descriptive figures of the NPC along with different biochemical agents see the recent comprehensive review by Jamali *et al.*, 2011 [18].

The nuclear pore complex (NPC; see Figure 1) is the exclusive gateway of material transport across the nuclear envelope (NE) [1], [2], [3]. This selective gateway plays a critical role in regulating transcription and protecting the genetic material of eukaryotic cells; consequently, its structure is highly conserved from yeast to vertebrates. However, the mechanism of transport across the NPC remains elusive and proposed models thus far remain incomplete and sometimes contradictory even for normal (wild-type) NPC [4], [5], [6], [7], [8], [9], [10], [11], [12].

The NPC is composed of about 30 distinct proteins collectively called nucleoporins (nups). Individual nups are directly related to several human diseases including influenza, cancers such as leukemia and inflammatory myofibroblastic tumors, and less frequent diseases like triple-A syndrome and primary biliary cirrhosis [13], [14], [15], [16]. Nups also play an important role in viral infections by providing docking sites for viral capsids, and also by blocking host cell mRNA export or inhibiting import of antiviral signals [17]. For a most recent comprehensive review about NPC-related disease see Jamali *et al.*, 2011 [18]. Along the same lines, Buehler *et al.*, 2010, have recently reviewed the role of protein mechanics in disease conditions [19].

**Figure 2:**
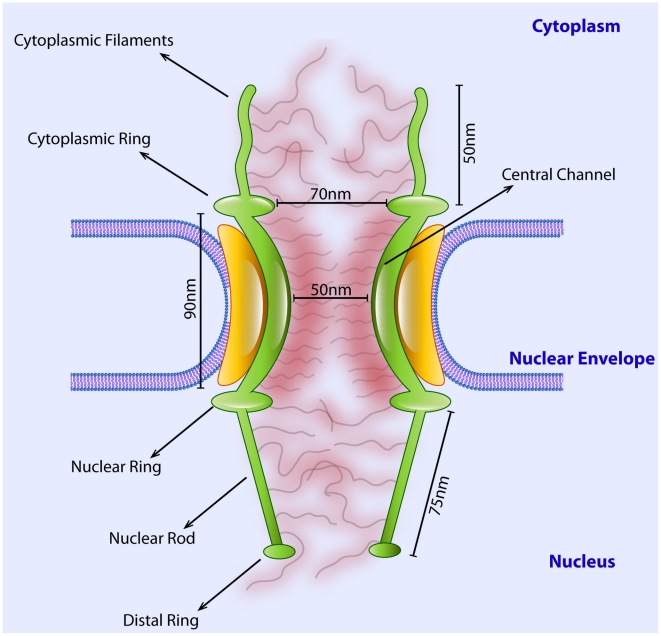
Dimensions of different subunits of the *Xenopus* oocyte NPC that were used in our model. Dimensions taken from Akey 1989 and Stoffler *et al.* 2003 [37], [38].

The total mass of the NPC is species-dependent and is about 44 MDa and 60 MDa in yeast and vertebrates, respectively [20]. The dimensions of the NPC also depend on the species. For example, yeast NPCs are 15% smaller than *Xenopus* NPCs [21]. The subunits of the NPC structure are cytoplasmic filaments, a cytoplasmic ring, a central channel that includes the spoke domains, a nuclear ring, and a nuclear basket that is composed of nuclear rods and a distal ring (see Figure 2 for the detailed dimensions of *Xenopus* oocyte NPC).

The NPC acts as a freeway for passive diffusion of molecules and ions smaller than ∼5 nm in diameter [22], [23], while actively controlling and facilitating the transport of larger cargos up to about 39–40 nm [7], [24] by discriminating between inert and karyopherin-bound cargos. Karyopherins (kaps) are a family of soluble proteins by which different cargos are shuttled between the cytoplasm and nucleoplasm via the NPC, in a process known as nucleocytoplasmic transport (NCT). Large cargo being imported/exported have to be bound to an appropriate kap in the cytoplasm/nucleoplasm via a signaling process to form the cargo-complex. A single NPC can accommodate a remarkable rate of transport on the order of ∼1000 translocations/sec, corresponding to a mass flow of ∼100 MDa/sec [7].

Importantly, almost 30% of nups include natively unfolded domains of phenylalanine-glycine (FG) repeats, collectively termed FG-repeat domains [25], [26]. It is believed that the key feature in nucleocytoplasmic transport is the interaction of kap with these natively unfolded repeat domains, and thus, FG-repeats are the central players in all proposed models. Indeed, distinctions between the different models originate from different speculations about the properties and spatial arrangement of FG-repeats within the NPC and the way they interact with kaps during the transport. While interacting with FG-repeats, kap escorts the cargo along the pore until it reaches the destination, where RanGTP dissociates the kap and the cargo is released [4].

The small size of the NPC and its compactness make it very hard to track individual cargo-complexes; hence, most studies are limited to bulk transport across many channels and cargos. However, a detailed understanding of the transport mechanism cannot be reached unless we examine individual cargos moving across the pore and inspect their interaction with surrounding nups, which can be genetically modified to model disease cases. While extensive efforts have been devoted to revealing the biochemical aspects of the transport mechanism, far less is known about its biophysical details.

Nucleocytoplasmic transport (NCT) happens in a channel about 50 nm wide and in a millisecond time scale *in vivo* [7], [27], [28]. Current imaging techniques fail to capture both the time and spatial resolution necessary to understand NCT. The refined resolution of single molecule imaging techniques become costly when we recognize their poor time resolution [29] and invasiveness, rendering them inappropriate for *in-vivo* measurement. Furthermore, even if they come close to these resolutions, they fail to capture transient interactions happening between FG-repeats and the cargo-complex on the order of nanoseconds. These uncertainties about the molecular events happening during nucleocytoplasmic transport (NCT) have been the source of contrasting speculations about the transport process across the pore.

Simulation models can probe into the narrow NPC channel to examine the sequential events leading to the NCT cycle. Molecular dynamics simulations are not applicable since both the NPC size and the transport time are beyond available computational recourses. However, molecular dynamic (MD) simulations have been used to examine the behavior of limited arrays of FG-repeats [30], [31], [32]. When it comes to the whole structure of the NPC, however, the best alternative to an all-atom MD is a coarse-grained model in which the atomic details are lost in order to obtain computational feasibility. Coarse-graining is known for its ability to study biological systems for time scales nearer to those in physiological conditions, though atomistic details are neglected [33].

Recently we showed that this methodology can be effectively employed to mimic the NPC functionality [34]. Here we present the details of the coarse-grained model of the unbiased functional state of the NPC along with new insights into nucleocytoplasmic transport. The model is based on an intensive literature survey on simulating biological phenomena via a coarse-grained approach and applying these methods to develop a coarse-grained model of the NPC. We look into the NCT mechanism at a fine resolution both in time and space to mimic the NPC from a biophysical perspective by implementing experimentally known data about the NPC and utilizing polymer physics principles. Once the model is established, we can use it to examine different hypotheses about transport as well as examining different factors that alter transport, i.e. changing molecular size, shape, hydrophobicity and so on. Former studies have been limited to a single sheet of FG-repeat being ruptured by a bead [35].

## Materials and Methods

**Figure 3:**
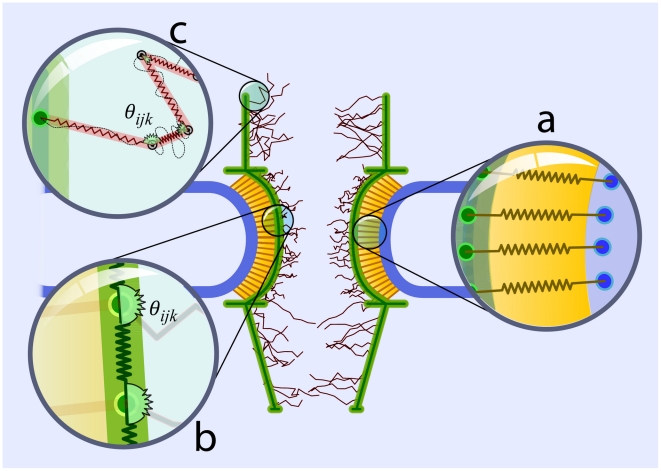
Our discretized model of the NPC structure includes three different groups of nups. **a**) Poms are responsible for anchoring the structure to the NE. They are modeled as a set of linear springs fixed at one end to the NE and at the other end to the central channel. **b**) The main scaffold includes cytoplasmic filaments, central channel, and nuclear basket, and is modeled by linear and angular springs. While the linear springs account for the elastic extension in the NPC backbone, angular springs explain the bending rigidity. **c**) FG-repeat domains are modeled as discrete wormlike chains (WLC) with the persistence length measured by AFM force-extension [39].

**Figure 4:**
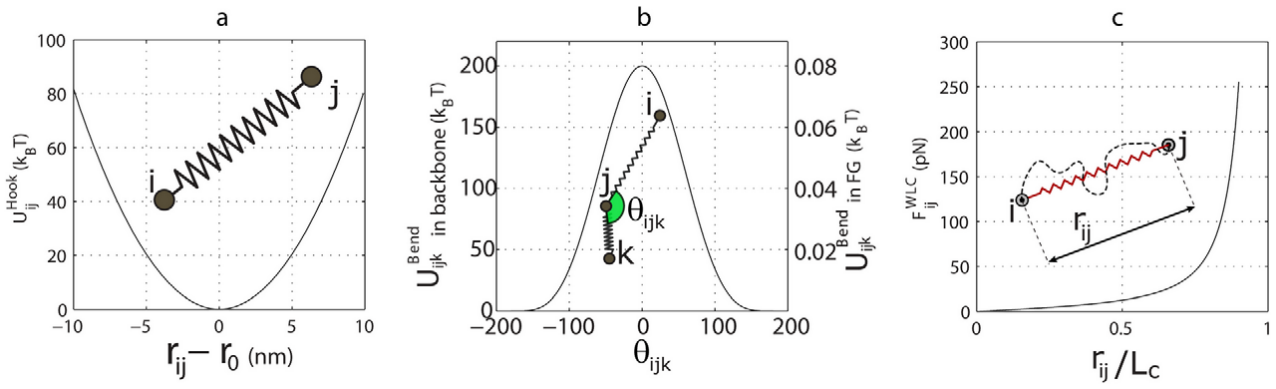
The energy and force-law of different springs between consecutive beads in the structural component of the NPC. **a**) Extensional elastic potential energy between two consecutive beads in the pom or main scaffold regions, representing the elastic extension. **b**) Angular potential energy between two consecutive springs representing bending rigidity of the main scaffold and the FG-repeat domains. Y-axis on the left shows values of the angular potential energy for the main scaffold, while the right y-axis shows those values for FG-repeat domains. **c**) The force-law of the wormlike chain (WLC). Discrete WLC models the FG-repeat domains.

Brownian dynamics simulations are carried out with 1 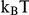 as the unit of energy, 1 nm as the unit of length, and 0.1 ns as the unit of time. All other parameters are reduced to dimensionless variables by using these units. Using the bead-spring model [36], the whole structure of the *Xenopus* oocyte NPC [37], [38], is discretized. The NE is treated as a rigid wall to which the central channel is anchored by a set of linear springs (see Figure 3-a). The NPC main scaffold, however, is considered to be elastic and discretized into linear springs (Figure 4-a):
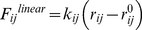
(1)where 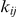 is the spring constant and 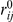 the equilibrium bond length.

In addition to the axial extension, the bending rigidity is taken into account by the following cosine-based potential energy between two consecutive segments (Figure 4-b):
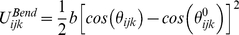
(2)where 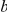 is the bending force constant and 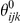 the equilibrium angle. This bending energy potential is applied to the main scaffold and FG-repeat domains.

For FG-repeats, axial extension is modeled by discrete wormlike chains (WLC) that are governed by the following force-law (Figure 4-c):
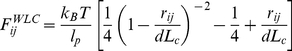
(3)where 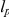 is the persistence length for FG-repeats [39], and 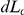 is the contour length of the segment.

**Figure 5:**
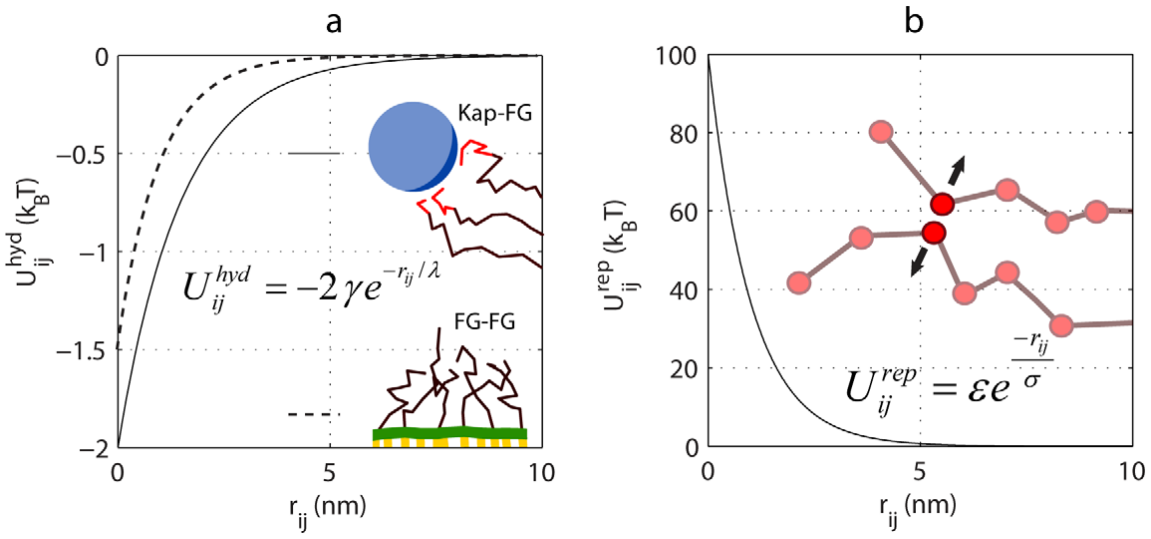
The nonbonded potential energies employed in the model. **a**) Hydrophobic potential energy is applied between FG-beads localized to the central channel (dashed line). The same potential, a bit stronger, is applied between kap and FG-beads (solid line). **b**) Pairwise repulsive potential.

The inter-FG as well as kap-FG hydrophobic affinity is modeled by the following long-range potential energy [40] with a cutoff radius of 10.0 nm (Figure 5-a):
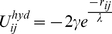
(4)where 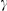 represents the hydrophobic affinity strength and is approximately 1.5–10 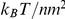 whereas the characteristic length 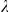 is 1–2 nm [40].

In addition, a short-range pairwise repulsive potential is applied between beads to avoid collision [41] with a cut-off radius of 1.35 nm (Figure 5-b):
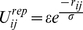
(5)where 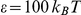, 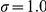 nm.

In the absence of inertial effects (i.e, diffusive regime or Stokes flow), the Langevin equations of motion are solved explicitly forward in time for every bead 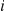 [42]:
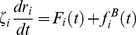
(6)where 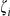 is the friction coefficient of bead 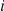 and obeys the Stokes' law for a spherical particle [43]: 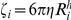, in which 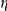 is the cellular viscosity and equal to ∼5 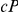 [44]. 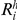, the hydrodynamic radius of the bead, is taken to be equal to the geometrical radius based on the average protein density and mass [45]. Therefore, for a bead 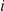 having a lumped mass of 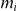, 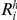 is 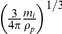 in which 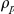 is the average density of the protein [45]. 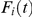 is the total conservative force acting on bead 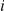 and 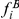 is the Brownian force with a Gaussian distribution and mean zero [43]. Integration of Eq. (6) leads to the following numerical equation of motion [42] (in x-direction):
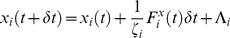
(7)

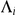
 is the random displacement due to the Brownian force and is independently chosen from a Gaussian distribution with a zero mean and a variance of 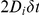, in which 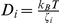 is the diffusion coefficient of the bead 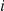, and 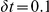 (dimensionless).

### The rationale for a coarse-grained model of the NPC

**Figure 6:**
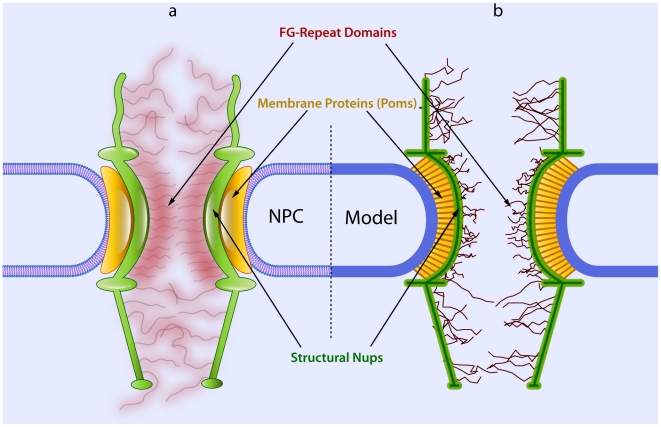
The structural components of the NPC and their equivalences in our model. **a**) The NPC structure is composed of three different groups of nups, namely poms (yellow), structural proteins (green), and FG-nups (red). **b**) In our model, we consider all these components with the appropriate set of bead-spring elements representing their *in-vivo* functions.

To develop an accurate coarse-gained model of the NPC, we need to consider its structural features. The NPC structure is composed of three different groups of nups (Figure 6-a), placed on top of each other [6], [46]:

1. Pore membrane proteins (Poms) that anchor the NPC scaffold to the NE and surround the central scaffold.
2. Structural proteins that give the NPC shape and strength and maintain the main scaffold.
3. FG-nups that provide the binding sites for NTRs and are central to selectivity.

In our coarse-grained model, we have considered all these groups with their corresponding functions (Figure 6-b). In the following, we discuss the rationales behind the assumptions of our model.

#### Pore membrane proteins

Poms are modeled as a set of parallel Hookean springs that on one end are fixed to the NE and on the other end are connected to the central channel, and therefore, do not allow the NPC to detach (Figure 3-a). This accurately represents the function of the poms in terms of anchoring the NPC to the NE. The elasticity of the spring elements is assumed equal to the average elasticity of the NE; that is, 17 kPa [47].

#### Structural proteins

The NPC main scaffold, consisting of the cytoplasmic filaments, central channel, and nuclear basket, is also assumed elastic [48], [49] and in our model is mimicked by bead-spring elements (Figure 3-b). Each bead in the NPC backbone represents a lumped mass of several kilodaltons. Spring constants, 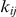
's in Eq. (1), are based on the elasticity of the NPC structure, while the equilibrium bond lengths, 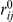
's, depend on discretization resolution as well as the dimensions of the NPC. Since to our knowledge there has not been any study on the material properties of the NPC structure, the elastic modulus of the NPC is considered equal to the mean value of that for the NE, 17 kPa. This seems reasonable because the NE is usually assumed uniform when its material properties are of interest [47], [50], [51]. However, this value is much larger than the 1–2 kPa elasticity of Nsp1 FG-hydrogel obtained by AFM [52], implying that the NE and hence the anchoring and structural nups are far stiffer than FG-nups.

In addition to the elastic extension in the backbone, we also consider the bending rigidity of the NPC with the bending force constant 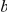 and the equilibrium angle 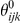 between bonds 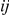 and 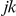 (Eq. (2) and Figure 3). Bending force constant is a material property, whereas the equilibrium angle depends on the NPC geometry and discretization. Again, due to the lack of information about NPC material properties, we arbitrarily choose the bending force constant equal to the minimum value that guarantees structural stability and does not let the disassembly of subunits during transport. This value was determined to be 100 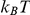.

#### FG-nups

As the third group of nups, FG-repeat domains are the innermost layer, and are believed to be the key players in the facilitated transport. They are natively unfolded domains [26] that adopt a string-like shape [6]. FG-repeats play the most important role in transport and hence in the following we cover them in more detail.

FG-motifs are scattered across the NPC structure and electron microscopy has localized FG-nups to the central channel as well as the cytoplasmic and nuclear faces [53]. In agreement with this, we include FG-repeats localized to both peripheries and inside the central channel.

In a recent AFM study, Lim *et al.* [39] suggested that the FG-repeat domains of nup153 adopt the wormlike chain (WLC) force-law (Eq. (2)) with an average persistence length of 0.43 nm [39]. This is an indication of their polymeric nature with a finite extensibility. Moreover, being tethered to the NPC at one end, FG-repeats dangle from the main scaffold into the peripheries and the confined space inside the pore. In harmony with these, we model the FG-repeat domains using beads that are connected by WLC springs. Each chain at one end is tethered to the main scaffold and, at the other end, dangles out and vibrates thermally (Figure 3-c).

Each bead in the discrete WLC represents an FG-motif. The total number of FG-motifs in our model is 720. Sine our model has two spokes, this is in agreement with the density of FG-motifs in the wild NPC, i.e., ∼2700–3000/NPC [46], [54]. It has also been found that the peripheral FG-nups are generally noncohesive, whereas the central FG-repeats are mainly cohesive [9]. The cohesion between central FG-domains is a result of the inter-FG hydrophobic interactions [7], [9], [52], [55], [56]. We take into account inter-FG hydrophobicity by incorporating the hydrophobic potential energy between FG-beads localized to the central channel (Eq. (4)). However, the peripheral FG-beads do not have such an affinity among themselves, allowing them to have higher mobility, and therefore, cover a wider space around the NPC periphery. This can potentially be an effective barrier for inert cargos, while increasing the chance of reaching to and pulling active cargo toward the NPC entrance.

The hydrophobic potential energy is long-range, and its parameters for FG-FG interactions are chosen such that the weak, transient interaction between FG motifs [7], [11], [57] is guaranteed. While the same potential form is applied for the kap-FG hydrophobic interaction, the choice of different parameters makes it possible to differentiate between FG-FG and kap-FG hydrophobic interactions. For that reason, the hydrophobic affinity strength 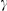 and the characteristic length 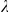 in Eq. (4) are respectively 2 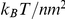 and 1.5 nm for kap-FG, whereas they are 1.5 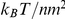 and 1 nm for FG-FG interaction. This is consistent with the observation that the overall FG-FG affinities are weak whereas the transport receptors, including kaps, are collectively more hydrophobic [9], [39], [55]. Moreover, these choices of 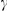 and 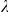 create an overall transient, weak hydrophobic interaction, so that the cargo-complex is not trapped within the FG-region and its dynamic motion is ensured [4], [6], [7], [27], [55], [57].

While the FG-beads serve as FG-motifs and interact with each other, with the solvent, and with cargo, the WLC springs represent the entropic effects of the FG-repeat domains. In fact, the WLC springs play the role of the hydrophilic linkers between FG-motifs. Hydrophilic linkers are a sequence of 5–30 charged amino acids between FG-motifs [58], [59] that hypothetically drive the FG-repeat regions into disorder [26]. A recent study suggests that these linkers contribute to the interactions between FG-domains [31]. Consistently, our model predicts that the polymeric elasticity of the discrete WLC combined with the thermal fluctuations dictates randomness to the FG-repeat domains and increases the chance of FG-motifs to meet and interact with each other.

Additionally, the persistence length (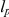) of the WLC serves as a measure of chain stiffness [60] and is assumed to be equal to 0.43 nm. This is the average value found by Lim *et al.* for the nup153 FG-repeats [39]. The contour length, on the other hand, is the maximum extendible length of the chain. In our model, we used the reported values for the fully extended length of different FG-repeat domains. Natively unfolded FG-domains are estimated to include ∼150–700 amino acid residues, which correspond to a fully extended length of about 50–200 nm [9]. Supposedly, the longer FG-repeat domains are mainly localized to peripheral nups [10]. For example, nup153 of the nuclear basket can be fully extended up to 180 nm [61]. Therefore, in this work we place the long FG-repeats (200 nm) in the periphery and the short FG-repeats (50 nm) in the central channel. This choice of lengths makes it possible for the FG-repeats to locally reach each other and span a wide area in the periphery, consistent with experimental results [9], [62].

Since we are using the discrete WLC, individual FG-repeats are discretized into several WLC segments, each with a corresponding contour length, 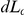 in Eq. (2). Discretization of each FG-repeat should be such that an individual WLC segment represents a large number of persistence lengths. This ensures that the discretization criterion of the WLC model in which each spring must be sufficiently larger than the persistence length is satisfied [63].

The finite-extensibility of the WLC imposes a singularity on its force-extension law, such that as the chain approaches the contour length, the axial force goes to infinity. This finite-extensibility is a well-known behavior of (bio)polymers. It has an important role in their rheology and is one of the key differences between the linear and non-linear springs [63]. From a numerical point of view, however, this might be a drawback because it can produce instability once the distance between two beads approaches the contour length. Specifically, this is likely to happen when an explicit time marching scheme is used in the framework of Brownian dynamics [63]. To overcome this flaw in our simulation, we use the rejection algorithm proposed by Öttinger [64], in which every movement of beads that leads to an artificial effect of extension near or beyond the contour length is rejected.

For the discrete WLC, it is also necessary to account for the bending potential energy between successive segments [63], [65], [66]. We use the same cosine-based bending potential energy that is used for the main scaffold (Eq. (2)); however, the bending force constant is three orders of magnitude smaller for FG-repeats due to their fine structure. This constant for a discrete WLC is directly related to the chain flexibility and therefore to the persistence length according to the following relation [65]:
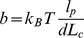
(8)where 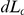 is the contour length of the segment. The bending force constant 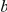 from this equation is then used in Eq. (2) for the discrete WLC.

In Eq. (2), the choice of the cosine-based potential energy for the bending rigidity of FG-repeats has some advantages. In addition to being computationally efficient [67] and numerically stable [68], the cosine-based potential energy works better for large fluctuations compared to the harmonic 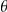 potential energy [67]. This is essential for an accurate modeling of the fine structure of FG-repeats, which undergo high degrees of fluctuations due to interaction with cargo and thermal noises.

The chain curvature in the WLC is based on thermal fluctuations [69] that are on the order of 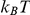. Our model consistently predicts that, in the course of transport, FG-domains show a high degree of flexibility exhibiting the character of natively unfolded proteins [26]. We found in our simulations that these fluctuations are the main barrier preventing inert cargo from entering the nucleus and hence the main factor in selective rejection.

Overall, the WLC is a powerful model to understand the properties of a wide range of synthetic and biological polymers [70] and numerous studies have been conducted using the WLC. It is indeed a valid assumption for individual FG-repeats, as was shown by Lim *et al.* [39]. However, the WLC falls short of including the effects of self-avoidance [70]. We overcome this physical flaw in our model by applying a pairwise repulsive potential energy between FG-beads (Eq. (5)). The repulsive potential energy also would reproduce the excluded volume effects [71] that are also absent in the WLC.

To replicate the excluded volume effects of polymer aggregations in a bead-spring model, several potentials, namely Lennard-Jones (LJ), Morse, and other exponential forms are used [72]. However, the soft potential in Eq. (5) has several advantages over the others. Namely, although LJ is commonly the choice for repulsive potentials, to avoid instability it requires a small time step in Brownian dynamics due to its steep behavior in short-distances [41]. This characteristic makes it computationally intensive and also violates the supposition of being in the diffusive regime (Stokes flow) of the intracellular environment. In this regime, inertial forces are neglected compared to viscous forces, a situation that is common to proteins within the intracellular environment [42]. Being in the diffusive regime means that particles lose their memories, and therefore, velocity relaxation time is much smaller than the time-step [43]. In simulation, this necessitates a selection of a large enough time-step to ensure it is far larger than the relaxation time. Using a soft repulsive potential like the one in Eq. (5) makes it possible to achieve this goal. Moreover, Eq. (5) is computationally more efficient compared to the Morse potential and has been shown to predict the polymer rheology better [41].

#### Cargo-complex

In addition to the structural features of the NPC, the cargo-complex and its interaction with the FG-repeats is another important factor that should be thought of in developing an accurate coarse-grained model. We consider the complex of NLS-cargo and kap-β as a solid sphere interacting with the solvent as well as the FG-repeat domains.

Kap-β has a boat-like shape [8], [46] that binds to the cargo on its concave surface while interacting with the FG-repeat domains on its convex surface [46], [73]. Crystallography [73], [74], [75], biochemical analysis [76], and molecular dynamics simulations [77] have revealed up to 10 hydrophobic binding spots on the convex surface of the kap-β.

**Figure 7:**
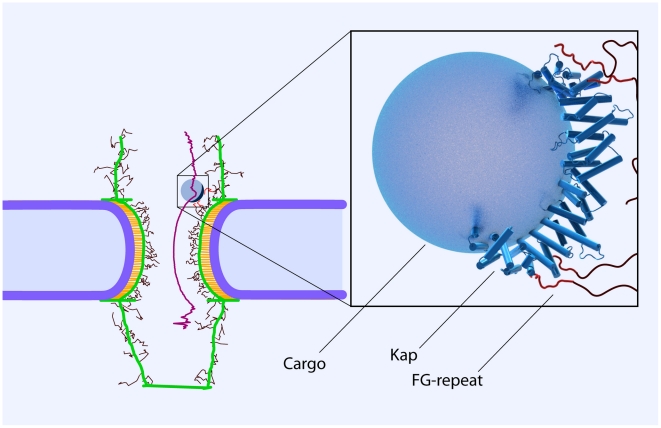
The cargo-complex interacting with FG-repeat domains via hydrophobic patches on the convex surface of the kap-β[46], [73]. Kap-β has a boat-like shape [8] and the localization of the binding spots on its surface has led to the idea of a “coherent FG-binding stripe” instead of discrete binding spots [46]. In our model, we take into account this fact by considering a ‘hydrophobic arc with limited capacity’ on the cargo surface. This arc possesses eight hydrophobic binding spots, and thus, is able to simultaneously interact with up to eighth FG-motifs. The magnification on the right shows a closer depiction of the cargo-complex with the crystal structure of the kap-β (blue) interacting with FG-repeats (red) on its convex surface (1F59, pdb bank). Also, the average path of a 15 nm cargo-complex during translocation is shown in purple. The path is averaged over 150 independent simulations. As it can be seen, the cargo-complex is primarily attached to the FG-layer during its translocation.

The localization of binding spots on the kap convex surface has resulted in the idea of a “coherent FG-binding stripe” instead of discrete binding spots [46]. In concurrence with this, we consider kap-β as a half-circle on the cargo-complex (Figure 7) with eight binding spots on its convex surface. This situation resembles a ‘hydrophobic belt with limited capacity’ (i.e., eight binding spots) on the cargo. In our model, all these binding spots have the same affinity, though it has been suggested that the various binding spots may have different affinities [54]. Kap-FG hydrophobic affinity is then mimicked by applying the hydrophobic potential energy introduced in Eq. (4) between binding spots of kap and FG-motifs lying within the cutoff radius. As noted above, we assume that the kap-FG hydrophobic affinity is a bit stronger than that of FG-FG. This is consistent with the fact that transport receptors are generally more hydrophobic compared to FG-domains [9], [39], [55]. Furthermore, to model steric repulsion and stop beads from penetrating into each other, the repulsive potential energy (Eq. (5)) is applied between the cargo-complex and every bead it approaches.

Different energy and force-laws along with the parameters that have been used in the model are shown in Table 1 and Figures 2 and 4. In addition to the above-mentioned force fields, there is no source of external energy or driving force in the model. This assures us that the transport *per se* in the model is not artificial in terms of energy, and there is no external force needed to make the cargo-complex pass the pore —a situation that is believed to be the case in biological conditions [4], [46], [61], [62], [78], [79]. This makes our model suitable for mimicking *in-vivo* experiments.

**Table 1:** Different values used in the coarse-grained model.

Parameter	Symbol/Formulation	Value	Reference(s)
Young's modulus of the NPC main scaffold	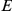	17 kPa	Estimated based on the [47]
Bending force constant of the NPC main scaffold	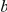	100 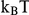	Estimated
Bending force constant of the discrete WLC	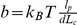	0.04 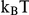	Estimated based on the [39], [63], [65], [66].
Contour length of the WLC: Peripheral FG-repeats	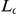	200 nm	Estimated based on the [9],[61]
Contour length of the WLC: Central FG-repeats	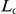	50 nm	Estimated based on the [9], [61]
Persistence length of the WLC	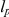	0.43 nm	[39]
Kap-FG hydrophobic affinity strength	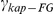	2 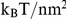	Estimated based on the [40]
Kap-FG hydrophobic characteristic length	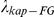	1.5 nm	Estimated based on the [40]
FG-FG hydrophobic affinity strength	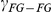	1.5 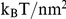	Estimated based on the [40]
FG-FG hydrophobic characteristic length	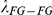	1 nm	Estimated based on the [40]
Density of protein	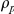	1.35 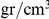	[45]
Cellular viscosity	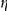	5 cP	[44]
Hydrodynamic radius: FG-beads	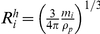	0.9 nm	Calculated based on the [43]
Hydrodynamic radius: main scaffold beads	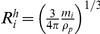	1.2–4.7 nm	Calculated based on the [43]
Drag coefficient: FG-beads	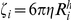	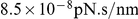	Calculated based on the [43]
Drag coefficient: main scaffold beads	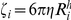	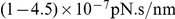	Calculated based on the [43]
Diffusion coefficient: FG-beads	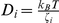	50 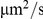	Calculated based on the [43]
Diffusion coefficient: main scaffold beads	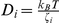	8–42 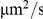	Calculated based on the [43]
Depth of bead-bead repulsive potential well	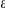	100 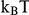	Estimated based on the [41]
Characteristic length of the repulsive potential	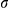	1.0 nm	Estimated based on the [41]

#### Computational cost

The computational cost of the model is about 15 CPU-hours per one millisecond of transport time. Moreover, approximately a constant memory resource of about 5 MB is required for each simulation. These data are calculated based on computing nodes at the San Diego Supercomputer Center (SDSC).

## Results

### Model validation: The first-passage time of a single active and passive cargo is consistent with the experimentally observed values

As the first step to validate our model, we explored the transport of a single, common-size cargo-complex 15 nm in diameter. The model's prediction of the mean transport time in the absence of molecular traffic and competing factors is in agreement with the experimental observations.

**Figure 8:**
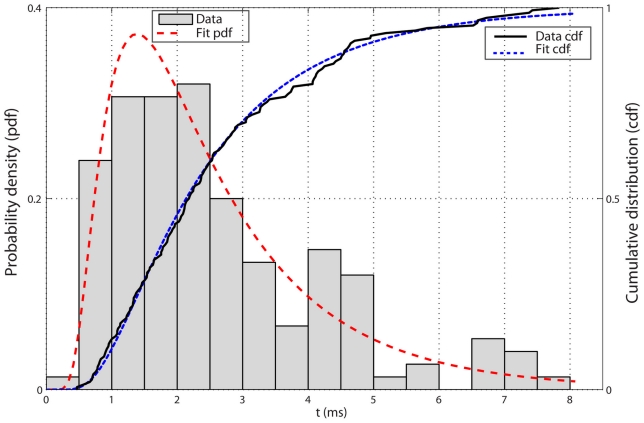
The mean transport time of a 15 nm diameter cargo complex is 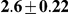 ms (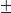
SEM) that is averaged over 150 independent runs. As it can be seen, the transport time is scattered over a wide range from 0.5 ms to 8 ms. The transport time can be viewed as the first passage time [80] if an absorbing wall is imagined in the nuclear compartment where the cargo is released. The histograms show the distribution of the first passage time that obeys the inverse Gaussian. Red dashed lines show the best fitted inverse Gaussian distribution with the scale parameter 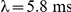 and the mean value 2.6 ms. Solid black line shows the cumulative distribution function (cdf) obtained from simulation results and blue dash lines represent cdf of the inverse Gaussian that is in a good agreement with simulation results.

To be statistically reliable, we ran 150 independent simulations, over which the first-passage time is averaged with the standard error of the mean (SEM) about 5%. Each run was continued until the cargo-complex was successfully imported into the nucleus. To save computational time, the cargo-complex is initially placed in the vicinity of the NPC entry, on the cytoplasmic side. The starting time of transport was defined as when the cargo-complex and FG-repeat domains in the cytoplasmic filaments interact for the first time. Accordingly, the end time was defined as when the cargo-complex passes the pore and is completely loaded into the nuclear basket for the first time. As soon as the cargo-complex is completely loaded into the basket, we assume that it is disassembled by interaction of RanGTP with kap, and therefore, the cargo is released and the simulation is ended. Under these conditions, our model predicts that the first-passage time of the cargo-complex is 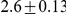 ms (mean 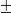
SEM, and hereafter). Distribution of the first-passage time is scattered over a wide range from 0.5 ms to 8 ms (see Figure 8), i.e. a 16-fold variation, which is an indication of the stochastic nature of nucleocytoplasmic transport. Based on our results, on average, 53% of transport time is spent in the central channel.

Next, we changed the cargo size and investigated the active transport of 9 and 20 nm cargo-complexes, independently. For each size, 50 independent simulations were carried out with the same conditions as aforementioned. We obtained the first-passage time of 9 nm and 20 nm cargo-complexes to be 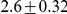 ms and 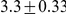 ms, respectively. For an in-depth study of the size effects on nucleocytoplasmic transport see [34].

Furthermore, we examined the capability of our model to conduct passive transport of small cargos (≤3 nm), which are known to diffuse freely across the NPC [22]. For the small cargo, we removed the hydrophobic affinity from its surface and ran 50 independent simulations under the same conditions as mentioned above. The first-passage time for the passive diffusion of 3 nm cargo was obtained to be 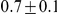 ms (Table 2).

**Table 2:** The different cargo-complex sizes.

**Cargo diameter (nm)**	9	15	20
**Diffusion coefficient ** ***D*** ** (** 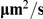 **)**	10.0	6.1	4.5
**Mean first-passage time ** 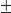 ** SEM (ms)**	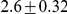	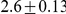	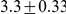
**Average number of participating binding spots during hydrophobic interaction**	7.90	7.89	7.90
**Average lifetime of a hydrophobic bond between single binding spot and FG-motif ** 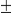 **SD (ns)**	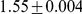	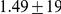	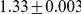
**Maximum bond lifetime of single binding spot-FG hydrophobic bond (ns)**	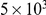	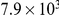	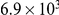
**Percentage of cargo-complex that shuttle back and forth at least once between the middle of the channel and cytoplasmic periphery**	36.0	7.3	10.7

### Distribution of the first-passage time obeys inverse Gaussian pattern with a positive drift

Our simulation results show that the first-passage time distribution of the cargo-complex obeys an inverse Gaussian distribution (Figure 8) with the following probability distribution function:
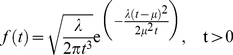
(12)where 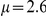 ms is the mean and 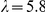 ms is the scale parameter.

Not coincidentally, this probability distribution function has been originally derived to predict the distribution of the first-passage time of a particle undergoing Brownian motion with a positive drift of 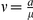, where 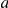 is the distance from an absorbing wall and 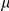 is the mean first-passage time [80]. In our model 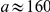 nm is the length over which the cargo-complex diffuses until it reaches the nuclear compartment (absorbing wall). Therefore, according to this distribution, in the absence of any molecular traffic and competing factors, a positive drift of 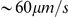 is expected for a single cargo-complex.

### The cargo-complex inside the central channel is primarily attached to the wall

**Figure 9:**
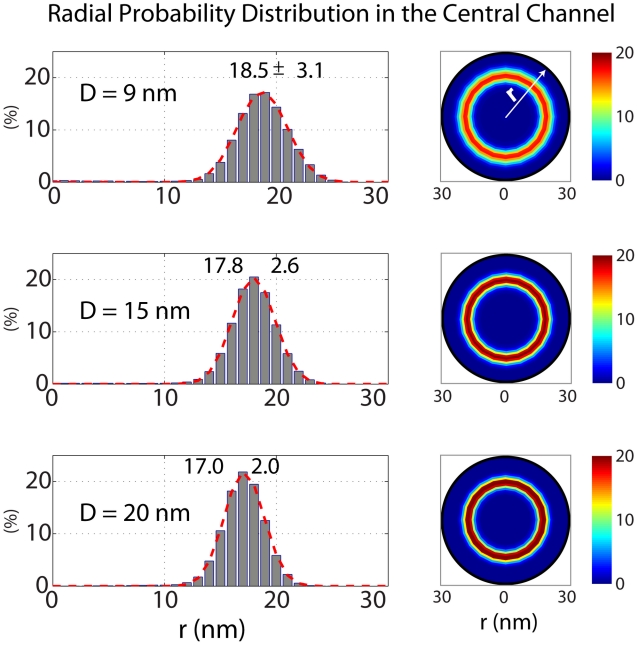
The histograms on left show the radial probability distribution of the cargo-complex versus the channel radius (the bin size is 1 nm). The cargo diameter is indicated inside the plot area. Each diagram is averaged over 50 independent simulations. Histograms are fit with Gaussian distributions (red dash line). The peak ± SD is recorded on top of histograms for each cargo size. Color bars on the right side show the radial probability distribution inside the central channel geometry. The more reddish, the higher the probability density. As it can be seen both in histograms and color bars, the large cargo is more likely to attach to the wall and less likely to disperse in the channel as opposed to the smaller cargo. This is partly due to its larger surface area and smaller diffusion coefficient, which make it less mobile compared the smaller cargo.

We analyzed the path of the cargo-complex in the central channel and averaged it over all independent simulations for each cargo size to find the radial probability distribution. The model suggests that inside the channel, the cargo-complex is most likely found near the wall, where it hydrophobically interacts with the FG-repeat layer on the wall. For a 15 nm cargo-complex, the radial probability distribution is maximized around 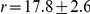 nm (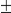
SD) and it rarely visits the channel axis. Similar results were obtained for 9 and 20 nm cargo-complexes (see Figure 9).

### The hydrophobic interaction between an FG-motif and a single binding spot on kap-β is highly transient

Using our model, we looked into the kap-FG hydrophobic interactions, which are believed to be intermittent and weak [7], [11], [57]. The model corroborates the highly transient nature of these bonds and suggests that the average lifetime of a hydrophobic bond between a single binding spot on the kap-β and an FG-motif during transport is approximately 1.5 ns with a standard deviation of about 17 ns. The minimum interaction lifetime is 0.01 ns (equal to the simulation time step), while its maximum is approximately 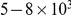 ns (Table 2).

### During transport almost all binding spots are engaged simultaneously in kap-FG hydrophobic interaction

The model predicts that during transport, almost all binding spots on kap-β simultaneously participate in the kap-FG hydrophobic interaction. In effect, once the cargo is hydrophobically engaged, on average about 7.89 out of eight binding spots are interacting simultaneously with FG-repeats (Table 2).

### Cargo-complex undergoes several back and forth fluctuations before exiting the NPC

**Figure 10:**
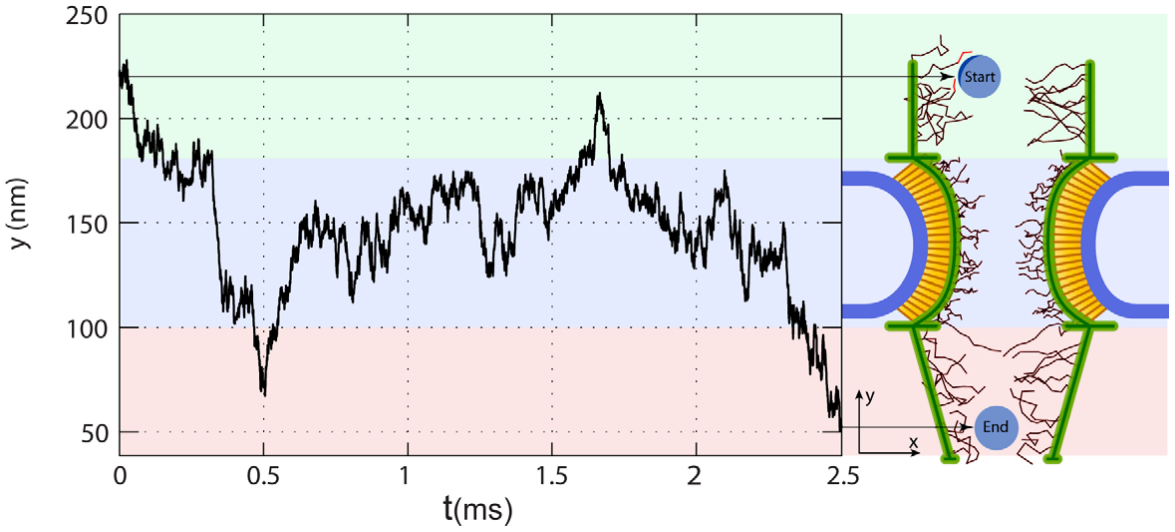
The y-component of a cargo-complex trajectory during ∼2.5 ms transport through the pore. The NPC structure is sketched on the right to illustrate the corresponding location of the cargo-complex within the NPC. It can be seen that the cargo-complex jumps back and forth tens of times before it released in the nuclear basket. The majority of its time is spent in the central channel.

Further, based on the cargo-complex trajectory during transport (Figure 10), we quantified back-and-forth fluctuations of the cargo-complex before exiting the NPC. The number of fluctuations is dependent on the length scale we are looking at. We found that 36% of 9 nm cargos, 7.3% of 15 nm cargos, and 10.7% of 20 nm cargos shuttle back and forth, at least once, between the NPC entry and the middle of the central channel.

### Cytoplasmic filament-deficient NPCs are far more permeable to inert cargos than intact NPCs

To shed light on the biophysical basis of the selectivity mechanism and the potential role of cytoplasmic filaments there, we investigated the diffusion of inert cargos across the NPC with and without cytoplasmic filaments.

First, in the intact NPC, we ran 50 independent simulations for inert cargo having a diameter of 15 nm, all with the same conditions as before. Next, we removed the cytoplasmic filaments from the NPC and carried out another 50 independent simulations with the same inert cargo. The only difference in these two sets of simulations, therefore, was the presence and the absence of cytoplasmic filaments. In both sets, each simulation was allowed to be run up to 8 ms, which is the maximum time an active cargo with the same size transported across the NPC (see Figure 8).

In intact NPCs, only 7% of inert cargos could diffuse through the NPC and reach the nuclear basket with an average time of about 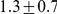 ms, while the rest (93%) were effectively inhibited and could not enter the central channel to reach its middle part. In NPCs lacking cytoplasmic filaments, however, the percentage of successful inert cargos significantly increased. Indeed, 46% of inert cargos could enter the central channel and diffuse to reach into the nuclear basket with an average time of 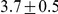 ms.

## Discussion

### The mean first-passage time is comparable with the dwell time that a single cargo-complex needs to traverse the NPC

The problem of the first-passage time [80] in our model becomes more clear if we imagine an absorbing wall in the nuclear compartment where RanGTP dissociates the kap from the cargo.

It should be noted that the first-passage time reported here does not take into account the time for the NLS-cargo and the kap to search for each other. This phenomenon has been suggested to increase the time between import cycles up to ∼10 s [11]. Moreover, our reported values do not include the time a cargo-complex needs to search for the NPC entry, because in our model the cargo-complex is initially placed near the cytoplasmic entry to save computational time. It also does not include the time for the cargo-complex disassembly in the basket as well as the time cargo needs to diffuse fully out of the basket and appear on the nuclear side. Therefore, it is important to note that there is a slight difference between the ‘first-passage time’ calculated herein and the ‘residence/dwell time’ reported experimentally in other works, which was found to be 1 to 9 ms depending on cargo and import agent concentrations as well as spatiotemporal resolution of their experimental setup [22], [27], [28], [81], [82], [83].

In the absence of molecular traffic and competitive factors, which for simplicity are ignored here, our model predicts that the mean first-passage time for a common-size cargo-complex of 15 nm is 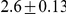 ms. This is about 20% more than the time required for the same particle to freely diffuse the corresponding distance of 160 nm in the cytoplasmic viscosity: 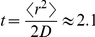 ms. The slight difference reveals that NCT is near the free diffusion regime. This would imply that the presence of FG-repeat domains might not anomalously affect the normal diffusion of the cargo-complex. Instead, it provides attractive binding sites, helping the cargo-complex pass through the NPC rather than repelling it away. Curiously, this also substantiates the common notion that the transport *per se* within the channel is energy-independent and does not need any motor protein [4], [46], [61], [62], [78], [79].

Furthermore, single molecular imaging techniques [27] have illustrated that the majority of the transport time is spent within the central channel. In our model, the same behavior is predicted and the cargo-complex spends approximately 53% of the total time in the channel, whereas the remaining time is shared between the cytoplasmic and nuclear compartments.

Interestingly, for a smaller cargo with the diameter of 9 nm the first-passage time is almost the same as 15 nm, whereas for a larger cargo of 20 nm a 27% increase is observed (Table 2). For a passive cargo of 3 nm in diameter, the mean first passage-time across the NPC is 0.7 ms, which is surprisingly 70% more than the same particle freely diffusing the same distance (0.4 ms). This is most likely due to the cramped structure of the NPC and the fact that passive cargo should pass through the confined channel.

### The cargo-complex slides over the channel inner wall

The model predicts that when the cargo-complex is passing through the central channel, it is mainly found near the wall where an FG-layer is formed. In effect, the long-range hydrophobic interaction between kap-β and FG-repeats is enough to attract the cargo-complex and keep it near the wall. Therefore, instead of randomly exploring inside the channel space, the cargo-complex ‘slides’ over the wall and diffuses back and forth until it reaches the nuclear basket. This scenario is an indication of the reduction-of-dimensionality phenomenon [84]. The idea is that reducing the dimensionality in which diffusion happens ensures a more rapid search toward the destination and thus enables a more efficient transport. It has been proposed elsewhere that nucleocytoplasmic transport is conducted by reduction of dimensionality [8], [46], which is also supported by our model. In line with this, remarkably, in a recent single-molecule imaging study the transport of cargo-free and cargo-bound kap-β was fluorescently tracked. Authors found that while inside the central channel, both kap-β and kap-β-cargo complex primarily locate near the channel wall and rarely occupy the axial channel [82]. Our results for different cargo sizes (Figure 9) corroborate their findings. While Figure 9 shows the radial probability distribution of the cargo-complex inside the central channel, in Figure 7 we plotted the mean trajectory of a 15 nm cargo-complex obtained from averaging over 150 independent transport simulations.

### Highly transient kap-FG hydrophobic bonds ensure an efficient translocation

Due to the hydrophobic affinity for FG-nups, the cargo-complex transiently binds to the FG-repeats and diffuses back and forth until it leaves the pore. Our model corroborates the highly transient nature of kap-FG interactions and predicts that a single hydrophobic bond between a binding spot on kap and an FG-motif persists, on average, only for about 1.5 ns. Indeed, during the millisecond event of NCT, millions of these fleeting interactions that are on the order of thermal noise happen between the kap and FG-regions. These super-fast interactions may explain the high transport rate (1000 translocations/NPC/s [7]) and the fact that the cargo-complex is not stuck inside the channel during translocation. This is in harmony with the perception that NCT is dependent on a sequence of relatively weak kap-FG interactions [4], [6], [7], [11], [57]. Interestingly, from this perspective the NPC can be imagined as a repository of billions of short-lived hydrophobic interactions per second. Nevertheless, rarely these weak bonds can last for up to several microseconds (see Table 2).

Importantly, during translocation when the cargo-complex interacts hydrophobically with FG-repeats, almost all of its binding spots simultaneously participate in interaction (see Table 2), which is partly due to the dense layer of FG-repeats. Experimentally, however, the number of binding spots that simultaneously participate in transport is still unknown [54]. Interestingly, the number of binding spots is directly related to the kap-FG avidity and our ongoing simulations (data not shown) show that this avidity has a biphasic effect on transport time: when there is no binding spot (inert cargo with zero avidity), transport is prohibited and the cargo is rejected; when there are too many binding spots (high avidity) the cargo-complex becomes trapped within the pore (see Text S1).

Additionally, the transitory kap-FG hydrophobic bonds bring about numerous back and forth movements of the cargo-complex inside the NPC. The shorter the length scale over which the cargo-complex is observed, the bigger the captured number of these jumps is. A meaningful length scale is the distance of the NPC entry to the middle of the channel. We found that less than half of the cargo-complexes undergo at least one back-and-forth in this region (Table 2). This means that once a cargo reaches the center of the channel it is more likely for it to reach into the nuclear basket than being aborted to the cytoplasm. This can put an upper-limit for abortive transports.

### Cytoplasmic filaments are mechanical components of the selectivity barrier

Our model suggests that cytoplasmic filaments play an important role in selectivity by repelling inert cargos and preventing them from entering the central channel. Upon removing these filaments in the model, the possibility of inert cargos to enter the central channel and passively diffuse across the NPC significantly increased (7% versus 46%).

When the inert cargo is in the cytoplasmic periphery it is repelled by cytoplasmic filaments and their FG-repeats. This implies the defining role of cytoplasmic filaments in the selectivity barrier. In effect, FG-repeats localized to cytoplasmic filaments tend to dangle out into the cytoplasmic area and sample it with their thermal motions. In this regard, the role of cytoplasmic filaments is significant because by their vacillating motions, they help cytoplasmic FG-repeats cover a wider area around the NPC entry. Therefore, through their thermally vibrated motions, these filaments, along with the attached FG-repeat domains, can effectively reject inert cargos.

Indeed, at the subcellular spatiotemporal scale, viscous forces are dominant compared to inertial forces [42], [85], and thus, in the absence of long-range hydrophobic affinity, an *‘impact’* by a cytoplasmic filament or their FG-repeats is enough to repel the inert cargo. It should be noted that the source of *impacts* at this scale is the steric repulsions between filaments and the cargo surface. This view is consistent with the virtual gate model, in which the peripheral FG-nups are suggested to act as “entropic bristles” that by their “push”, keep inert cargos away from the central channel [6].

In addition to repelling inert cargos, cytoplasmic filaments provide initial docking sites for cargo-complexes, as proposed elsewhere [86], [87], and possibly direct them toward the channel entrance (see Video S1). Importantly, this implies the possible role of these filaments in transport efficiency by making an *‘attractive trap’* for active cargos while constructing an *‘entropic barrier’* to inert cargos. This means that these filaments are not only essential for rejecting inert cargos, but are also likely to contribute to an efficient transport of active cargo.

From an active transport perspective, there are two common viewpoints about the functional role of cytoplasmic filaments. The first is that these filaments along with their pliable structure provide the initial docking sites for import complexes and thereby increase the transport efficiency [86], [87]. Along the same lines, it has been recently suggested that cytoplasmic filaments increase the “capture area” for active cargos in the cytoplasmic periphery [88]. The second hypothesis is that they are dispensable for kap-α/β-dependent import because the import of NLS-cargo shows a slight reduction in cytoplasmic filament-deficient NPCs [89]. From an inert cargo point of view, however, to our best knowledge, there has been no study so far on the potential role of these filaments in inhibition of inert cargo transport. Here, we propose that cytoplasmic filaments along with their localized FG-repeat domains are *‘mechanical components’* of the selectivity barrier. In other words, while the inner channel might be sufficient to create *biochemical* selectivity, our results show the necessity of cytoplasmic filaments in explaining *mechanical* selectivity.

### Conclusion and future directions

Our model is intended to be a platform to examine transport hypotheses and to suggest a plausible transport mechanism by scrutinizing these hypotheses *in silico*. At the first attempt, we have suggested elsewhere that the NPC works as a “lubricated gate” [34]. The model can also be used as a platform to study mechanics of nups in NCT and related disease, since the protein mechanics has been suggested to have a role in disease conditions [19].

Detailed understanding of the NCT mechanism may allow for development of a new set of drugs that work by intervening in nucleocytoplasmic transport. Many current drugs work by manipulating transport channels that are located on the cell membrane. We can envision a next generation of drugs based on manipulating transport across the nuclear membrane. To design such drugs, as a barrier against viral infection and to modulate gene expression, a thorough understanding of NCT mechanism is necessary. Recent development in virtual patient models have been used in the pharmaceutical industry to accelerate drug testing [90], and we believe that we need a virtual test tube to examine NCT.

Moreover, some open questions can be investigated using the coarse-grained approach that otherwise are not reachable by biochemical experiments. An important question in this realm is about the unique geometry of the NPC structure and its plausible role in transport. What is the biological explanation behind the hourglass shape of the central channel? What advantages does this specific shape give to the transport of a cargo-complex? While there is symmetry around the planes perpendicular to the NE, why is there an asymmetry relative to the NE, i.e. why are the cytoplasmic and nuclear peripheries different? Does this asymmetry have a role in transport? Indeed, the answers to these questions await further investigation.

In this regard, a more interesting question is: how does the complicated functionality of a large-scale proteinous complex (here, the NPC) emerges from simple elements (here, individual nups)? This kind of biological paradigm is receiving attention in literature [91].

On the other hand, of main limitations to a coarse-graining approach to the NPC, is poor information about the structural properties of the NPC and its building block, i.e. nups. This limitation can be addressed by experimental studies measuring material properties of the NPC under the same physiological conditions inside the cell.

## Supporting Information

Text S1 On the cargo transport through the NPC: Analysis of mean square displacement and the biphasic effect of the kap-FG avidity.
(DOCX)

Video S1 Nucleocytoplasmic transport of a 15-nm cargo-complex through the NPC.
(SWF)
