# Supplementary material for: Brownian Dynamics Simulation of Nucleocytoplasmic Transport: A Coarse-Grained Model for the Functional State of the Nuclear Pore Complex
Source: PLoS Comput Biol. 2011 Jun 2;7(6):e1002049. doi: 10.1371/journal.pcbi.1002049 (PMC3107250; doi:10.1371/journal.pcbi.1002049)
Supplement: Text S1 — On the cargo transport through the NPC: Analysis of mean square displacement and the biphasic effect of the kap-FG avidity. (DOCX) [file pcbi.1002049.s001.docx]

**A quick view into the cargo transport: Effect of size, mean square displacement, and the kap-FG hydrophobic avidity**

Ruhollah Moussavi-Baygi, Yousef Jamali, Reza Karimi^1^, Mohammad R.K. Mofrad*

Molecular Cell Biomechanics Laboratory, Department of Bioengineering, University of California, Berkeley, CA

*****Corresponding Author: [mofrad@berkeley.edu](mailto:mofrad@berkeley.edu)

**On effects of the cargo size on transport:**

To shed light on the effects of cargo size on transport, we measured the first passage time of cargo-complexes having sizes between 9 nm and 20 nm. We furthermore, investigated the 23 nm cargo-complex. Interestingly, for all sizes less than or equal 15 nm, the average of the passage time is approximately the same and equal to 2.6 ms. However, beyond this size, the passage time begins to increase continuously. Figure S1 shows how passage time scales with cargo size.

For each size, we calculated, analytically, the time required for the same particle to freely diffuse the same distance it travels through the NPC. Curiously, smaller cargos (<= 15 nm) have bigger differences with their free diffusion time. For example, the passage time of the 9-nm cargo-complex is 100% more than its free diffusion, whereas the passage time of the 20-nm cargo-complex is only about 18% longer than free diffusion (see Figure S2). It seems that for the larger cargo-complexes, transport is mainly governed by the cargo-size, and therefore the passage time is close to that in free diffusion. On the contrary, for smaller cargo-complexes, transport is almost independent of the cargo size and is mainly governed by kap-FG hydrophobic interactions. We therefore speculate that for large cargo-complexes, transport is ‘diffusion-limited’, whereas for small cargo-complexes transport is ‘interaction-limited’.


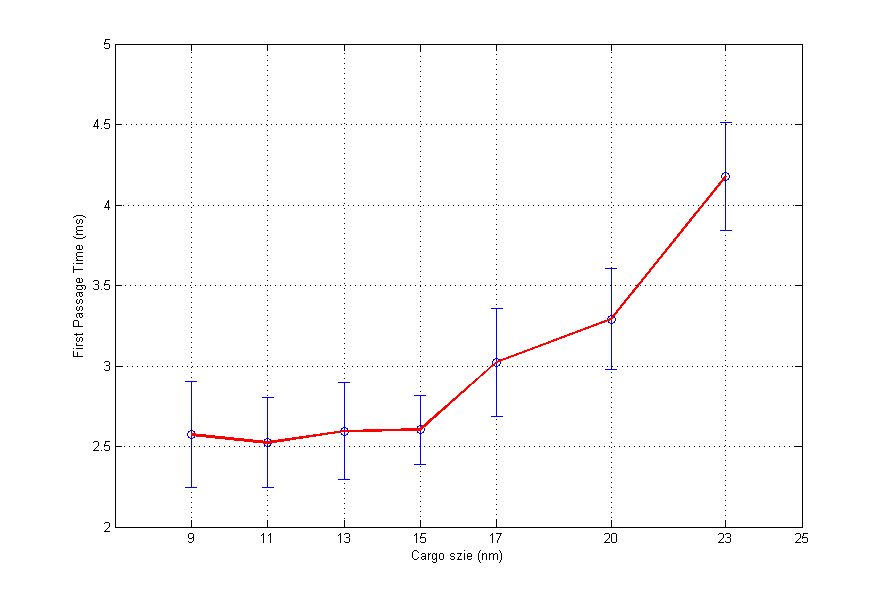


Figure S1. The variation of first-passage time vs cargo size.

Figure S2. The percentage increase of passage time compared to free diffusion

**Analysis of mean square displacement of the cargo-complex inside the NPC:**

Mean squared displacement (MSD) of the cargo-complex was calculated based on average value for each delta t, computed across all possible time intervals and all simulation sets for the 15 nm cargo-complex. The results are displayed in Figure S3 and are compared with corresponding values for free diffusion. Apparently, in small time scales the free diffusion holds while for larger time scale the confinement effect are evident. For the axial direction (y or the transport direction), the MSD is almost a flat line with a lower slope indicating an effective diffusion only slightly lower than the free diffusion. However, for the radial direction, MSD values are abruptly lower at large time scales due to confinement effects. The reduced diffusion in radial direction is consistent with a recent observation by Lowe et al., 2010 [[1](#_ENREF_1)]. These lower values are a direct effect of steric forces applied by walls to confine the particle within the channel.

**
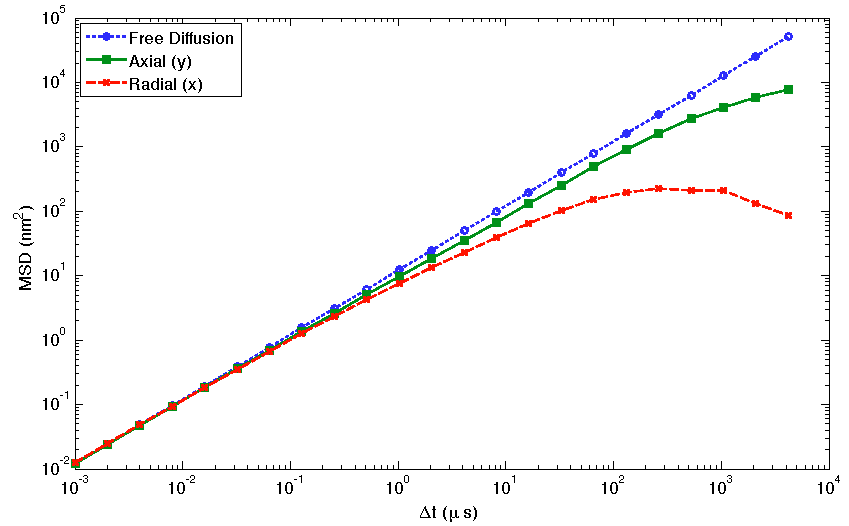
**

Figure S3. MSD values for axial and radial direction compared to free diffusion.

**The biphasic effect of the kap-FG avidity on transport time:**

When the avidity between cargo and the FG-repeat domains is zero, i.e., when there is no transport receptor (resembling the inert cargo), the transport is prohibited by cytoplasmic filaments (see the main text). In this situation it can be said that the mean first passage time goes to infinity. On the other extreme, when the number of binding spots on the transport receptor surface increases enormously, the avidity between cargo-complex and FG-domain intensifies greatly.Our ongoing simulations show thatin this situation the cargo-complex stuck in the NPC and thus the passage time increases significantly(see Figure S4). This biphasic behavior can be seen in the following graph. Note that our data is not complete for the high number of binding spots. This is because as the number of binding spots increases, the simulation time increases dramatically.

Figure S4. The biphasic effects of the kap-FG avidity on the passage time

**References of Text S1**

1. Lowe AR, Siegel JJ, Kalab P, Siu M, Weis K, et al. (2010) Selectivity mechanism of the nuclear pore complex characterized by single cargo tracking. Nature 467: 600-603.
